# Application of data mining to intensive care unit microbiologic data.

**DOI:** 10.3201/eid0503.990320

**Published:** 1999

**Authors:** S A Moser, W T Jones, S E Brossette

**Affiliations:** The University of Alabama at Birmingham, 35233-7331, USA. moser@uab.edu

## Abstract

We describe refinements to and new experimental applications of the Data Mining Surveillance System (DMSS), which uses a large electronic health-care database for monitoring emerging infections and antimicrobial resistance. For example, information from DMSS can indicate potentially important shifts in infection and antimicrobial resistance patterns in the intensive care units of a single health-care facility.


### Application of Data Mining to Intensive Care Unit Microbiologic Data[1]

**Stephen A. Moser, Warren T. Jones, and Stephen E. Brossette**
The University of Alabama at Birmingham, Birmingham, Alabama, USA

We describe refinements to and new experimental applications of the Data Mining Surveillance System (DMSS), which uses a large electronic health-care database for monitoring emerging infections and antimicrobial resistance. For example, information from DMSS can indicate potentially important shifts in infection and antimicrobial resistance patterns in the intensive care units of a single health-care facility.

We have defined a new exploratory data mining process for automatically identifying new, unexpected, and potentially interesting patterns in hospital infection control and public health surveillance data. This process, and the system based on it, Data Mining Surveillance System (DMSS), use association rules to represent outcomes and association rule confidences to monitor changes in the incidence of those outcomes over time. Through experiments with infection control data from the University of Alabama at Birmingham Hospital, we have demonstrated that DMSS can identify potentially interesting and previously unknown patterns. Future work on prospective clinical studies to determine the usefulness of DMSS in hospital infection control is needed, as is improved event presentation for the user and strategies for handling larger datasets.

The statistical strategies developed for automatically detecting temporal patterns in surveillance data require that analysts explicitly define outcomes of interest before surveillance begins. The Data Mining Surveillance System (DMSS), on the other hand, is not constrained to monitoring changes in user-defined outcomes. In DMSS, complex outcomes are represented by association rules, and outcome incidence is captured monthly.

An early version of DMSS, along with association rules and early experiments with a single organism, has been described (1). We briefly describe a newer version of DMSS and experimental results obtained by using it to analyze 1 year's data from intensive care units (ICUs) at the University of Alabama at Birmingham Hospital.

DMMS uses the following definitions. An itemset is a subset of the set of all items. The support of an itemset $x$, sup $(x)$, is the number of records that contain $x$. If sup $(x) \geq$ FSST, where FSST is the frequent set support threshold (FSST), then $x$ is a frequent set. An association rule, $A \Rightarrow B$, where A and B are frequent sets and $A \cap B = \varnothing$, is a statement about how often the items of B are found with the items of A. The incidence proportion of $A \Rightarrow B$, denoted ip($A \Rightarrow B$), is equal to sup($A \cup B$)/sup($A$). The precondition support of association rule $A \Rightarrow B$ is sup(A). The incidence proportion of an association rule $A \Rightarrow B$ in data partition $p_i$ describes the incidence of the outcome, B, in the group, A, during time $t_i$. A series of incidence proportions for $A \Rightarrow B$ from partitions $p_1, p_2, \ldots, p_n$ describes the incidence of the outcome B in group A from $t_1$ through $t_n$. Therefore, by analyzing the series of incidence proportions of an association rule $A \Rightarrow B$, it should be possible to detect important shifts or trends in the incidence of B in A over time. In this way, surveillance of B in A is possible.

Bacterial susceptibility and related demographic data of patients in the University of Alabama at Birmingham Hospital ICUs (medical, surgical [SICU], cardiac, neurologic [NICU]) during 1997 were extracted from the PathNet laboratory information system. Each record describes a single isolate and contains the following data elements: date of admission, date

Address for correspondence: Stephen A. Moser, University of Alabama at Birmingham, Department of Pathology, P246, 619 19th St., South Birmingham, AL 35233-7331, USA; fax: 205-975-4468; e-mail: moser@uab.edu.

[1]Presented in part at the International Conference on Emerging Infectious Diseases, March 8-11, 1998, Atlanta, Georgia.

of sample collection, date of results reported, source of isolate (e.g., sputum, blood), organism isolated, organism Gram stain and morphologic features, patient's location in the hospital, and resistant (R), intermediate (I), or susceptible (S) test results to relevant antibiotics, according to the National Committee for Clinical Laboratory Standards MIC breakpoints (2).

Duplicate records were removed so that for each patient, no more than one isolate per organism per month was included. In each remaining record, certain antimicrobial drug items were removed (only drugs to which the organism is historically susceptible at least 50% of the time remained). Additionally, items of the form S~Antimicrobial were removed so that only I~Antimicrobial and R~Antimicrobial items remained. Finally, data were divided into 1-month partitions ($p_1 \ldots p_n$) before analysis. For each partition $p_i$, all frequent sets with support of at least 3 (FSST >2) and association rules with precondition support greater than 5 were generated. Both the frequent set discovery and association rule-generating algorithms are beyond the scope of this review (3).

Each generated association rule must pass a set of rule templates that describe families of interesting and uninteresting rules. Each template is a construct of the form $be_1 \Rightarrow be_2$, where $be_1$ and $be_2$ are Boolean expressions over items and attributes. Association rule $A \Rightarrow B$ satisfies rule template $be_1 \Rightarrow be_2$ if A satisfies $be_1$ and B satisfies $be_2$. Two types of association rule templates are used: include templates and exclude templates. An association rule $A \Rightarrow B$ passes a set of rule templates if $A \Rightarrow B$ satisfies at least one include template in the set and does not satisfy any exclude template in the set.

Rule templates are handcrafted by domain experts to eliminate inherently uninteresting or nonsense rules. This is accomplished through iterative experiments with representative data by initially using few templates and then creating and modifying templates on the basis of pattern review.

History is a database that holds association rules and their incidence proportions for different data partitions. In DMSS, the user specifies a set of rule templates that contains any number of inclusive and restrictive templates (Table 1). Only association rules that pass the rule templates are included in the history. To establish a baseline for an association rule, the incidence proportions of the rule for the three previous partitions are obtained and stored in the history. Once stored in the history, a rule is updated for each new partition regardless of whether or not it is generated in the partition. Therefore, for every association rule, the history contains an up-to-date time-series of incidence proportions.

By analyzing information stored in the history, DMSS generates alerts that describe an extreme change in the incidence of an outcome B in a group A over time. For example, Table 2 describes the incidence of *Acinetobacter baumannii* in a nosocomial tracheal aspirate and in SICU isolates over the past six partitions. Clearly, a shift in incidence occurs between the first 4 months and the most recent 2 months of the series. If we call months 1, 2, 3, and 4 the past window, $w_p$, and months 5 and 6 the current

Table 1. Templates used to filter association rules

| Template type | Left ($be_1$) | Right ($be_2$) | Explanation |
|---|---|---|---|
| Exclude | (R~Antibiotic) | (Anything) | Want antibiotic sensitivity info on the right only. |
| Exclude | (Anything) | (Source) | Source of infection is not an outcome. Therefore, exclude all rules with a source on the right. |
| Exclude | (NS OR Org OR GrMP) | (NS OR Org OR GrMP | NS, Org, and GrMp are more informative if kept together in either a group or an outcome. |
| Exclude | (Loc) | (Org OR GrMp) AND (R~Antibiotic) | If the left contains location, then exclude rules that have Org and R~Antibiotic or GrMp and R~Antibiotic. |
| Include | (Org OR Loc) | (R~Antibiotic OR GrMp OR Org) AND Not(Loc) | Include rules whose groups are Org- or Loc-specific and whose outcomes are Antibiotic- or GrMp-specific. |

$be_1$ and $be_2$, Boolean expressions; R, resistant; NS, nosocomial; OR, "or"; Org, organism; GrMp, Gram stain and morphology; Loc, Location.

Table 2. A sample event generated by the Data Mining Surveillance System

| Association rule | | | $p_{c-5}$[a] | $p_{c-4}$ | $p_{c-3}$ | $p_{c-2}$ | $p_{c-1}$ | $p_c$ |
|---|---|---|---|---|---|---|---|---|
| (nosocomial, SICU[b], tracheal aspirate} | ==> | {*Acinetobacter baumannii*} | 0/11 | 0/10 | 0/9 | 0/13 | **2/9** | **3/9** |
| | | | | $w_p$[c] | | | $w_c$ | |

[a]$P_c$, current pair.
[b]SICU, surgical intensive care unit.
[c]$w_p$, past window; $w_c$, current window.

window, $w_c$, we can ask if there is an extreme change in the incidence between $w_p$ and $w_c$. We compute the cumulative incidence proportion for $w_p$ (0/43) and for $w_c$ (5/18) and compare the two by a statistical test of two proportions. To generate an alert for an association rule r, DMSS first constructs a current window ($w_c$) and a past window ($w_p$) on the series of incidence proportions of r ($w_c[r,0]$, $w_p[r,0]$ from the algorithm in the Figure). Second, it computes the cumulative incidence proportion for each window. Third, it compares the two cumulative incidence proportions by a test of two proportions. Finally, if the difference between the proportions is statistically extreme ($p \leq \alpha = 0.01$), it generates an alert. The value of α is user-defined and rather arbitrary. If an alert is not generated, the next set of current and past windows is formed ($w_c[r,1]$, $w_p[r,1]$ from the algorithm in the Figure), and the cumulative incidence proportions are compared. Window pairs are generated for the same association rule until an alert is generated or no more window pairs remain to be formed. DMSS generates all alerts by executing the procedure described on every association rule in the history.

i=0; k=0
while ($p_{c-2i-1}$ exists for *r*){
  j=0
  while ($p_{c-2i-1-j}$ exists for *r*){

$$w_c[r,k] = \bigcup_{n=0}^{i} p_{c-n}$$

$$w_p[r,k] = \bigcup_{n=0}^{i+j} p_{c-n-i-n-1}$$

   j++; k++
  }
  i++
}

Figure. Algorithm used to construct current and past windows for association rule r.

Current and past window pairs are generated by the algorithm in the Figure. If n is the number of incidence proportions in the history for a given rule, ($w_c$:$w_p$) pairs are generated for that rule in the following order: $(p_c{:}[p_{c-1},p_{c-2}]\}),\ldots,(p_c{:}[p_{c-1},\ldots,p_{c-n}]),([p_c,p_{c-1}],[p_{c-2},p_{c-3}]\}),([p_c,p_{c-1}]\},[p_{c-2},p_{c-3},p_{c-4}]),([p_c,p_{c-1}],[p_{c-2},p_{c-3},p_{c-4},\ldots,p_{c-n}])$, $([p_c,p_{c-1},p_{c-2}],[p_{c-3},p_{c-4},p_{c-5}]\}),([p_c,p_{c-1},p_{c-2}]\},[p_{c-3},p_{c-4},p_{c-5},p_{c-6}]\}),\ldots,([p_c,p_{c-1},p_{c-2}]\},[p_{c-3},p_{c-4},p_{c-5},p_{c-6},\ldots,p_{c-n}])$. For each pair, $w_p$ must be at least as large as $w_c$.

The total number of events was reduced from 251, by including all rules, to 36, by using the templates in Table 1; thus, classes of inherently uninteresting rules were eliminated. A retrospective look at the 155 events eliminated by the rule templates showed that they were uninformative. Therefore, the introduction of templates resulted in a more focused presentation of DMSS output.

Of the 36 events, 18 were judged potentially interesting. Table 3 contains several representative events, one per row. Each row contains the association rule, the incidence proportions in $w_c$ (bold), and the incidence proportions in $w_p$ (nonbold). For example, event 1 in Table 3 describes an increase in the number of *Staphylococcus aureus* resistant to oxacillin, clindamycin, and erythromycin isolated from tracheal aspirates in the fourth partition, and compared with those isolated in the 2nd and 3rd partitions. Of the events identified by DMSS, only the NICU and SICU had events that were location-specific (Table 3), while eight events were not.

The events identified by DMSS must be investigated by domain experts to determine

Table 3. Representative events identified and considered of potential interest

| Left Denominator | | Right Numerator | Partition 1 | 2 | 3 | 4 | 5 | 6 | 7 | Interpretation |
|---|---|---|---|---|---|---|---|---|---|---|
| *Staphylococcus aureus* Source TRACHASP[c] | ==> | R~Oxacillin[a,b] R~Clindamycin R~Erythromycin | | 0/10 | 0/8 | **7/14** | | | | Increase in the incidence of oxacillin (ORSA), clindamycin, and erythromycin resistance in all *S. aureus* isolated from tracheal aspirates. |
| NSNoso[d] | ==> | R~Ceftazidime | | | | 3/88 | **11/70** | | | Increase in incidence of ceftazidime resistance in all nosocomial isolates. |
| NP_GNR[e] | ==> | R~Piperacillin | | | | 0/17 | **6/14** | | | Increase in the LocSICU incidence of piperacillin resistance in non-pseudomonas gram-negative bacilli isolated from NSNoso. |
| NP_GNR | ==> | R~Piperacillin | | | 1/12 | 0/14 | **4/11** | **4/8** | | Increase in the LocSICU[f] incidence of piperacillin resistance in non-pseudomonas, nosocomial, gram-negative bacilli from the SICU. |
| NSNoso LocNICU[g] | ==> | *S. aureus* | 3/26 | 3/26 | 2/28 | **6/27** | **5/20** | **3/11** | | Increase in the incidence of nosocomial *S. aureus* in nosocomial isolates from the NICU. |

[a]R, resistant.
[b]Oxacillin, resistance implies resistance to amoxycillin/clavulanic acid, cephalothin, and cefazolin.
[c]SourceTRACHASP, tracheal aspirates.
[d]NSNoso, nosocomial (3 days from admission).
[e]NP_GNR, non-pseudomonas gram-negative rod.
[f]LocSICU, location, surgical intensive care unit (SICU).
[g]LocNICU, location, neonatal intensive care unit (NICU).

their actual importance. In this example, the data burden was small since in a prospective analysis only a few events would be presented to the user each month, thus allowing for the investigation of each event.

We believe that this approach to surveillance will allow hospital infection control programs to focus their limited resources on issues of probable significance. We also believe that this approach is a step toward the public health surveillance system described by Dean, Fagan, and Panter-Conner (4).

This work was supported in part by cooperative agreement U47-CCU411451 with the Centers for Disease Control and Prevention (SAM) and a predoctoral research fellowship LM-00057 from the National Library of Medicine (SEB).

Dr. Moser is associate professor, Department of Pathology, University of Alabama at Birmingham, and serves as director of Laboratory Information Services, associate director of Clinical Microbiology for University Hospital, and director of the Pathology Informatics Section. His research interests are applied research in diagnostic microbiology and the application of software as an aid to the intelligent analysis of medical information, especially that generated in laboratory medicine.

### References

1. Brossette SE, Sprague AP, Hardin JM, Waites KB, Jones WT, Moser SA. Association rules and data mining in hospital infection control and public health surveillance. J Am Med Inform Assoc 1998;5:373-81.
2. National Committee for Clinical Laboratory Standards. Methods for dilution antimicrobial susceptibility tests for bacteria that grow aerobically. 4th ed. Approved standard. NCCLS document M7-A4. Wayne (PA): The Committee; 1997.
3. Brossette SE. Data mining and epidemiologic surveillance [dissertation]. Birmingham (AL): University of Alabama at Birmingham; 1998.
4. Dean AG, Fagan RF, Panter-Conner BJ. Computerizing public health surveillance systems. In: Teutsch SM, Churchill RE, editors. Principles and practice of public health surveillance. New York: Oxford University Press; 1994. p. 200-17.

